# Vascular lipidomics analysis reveales increased levels of phosphocholine and lysophosphocholine in atherosclerotic mice

**DOI:** 10.1186/s12986-022-00723-y

**Published:** 2023-01-04

**Authors:** Li Zhang, Liling Xiong, Li Fan, Haoyang Diao, Mi Tang, Erdan Luo, Wenmei Guo, Xiao Yang, Shasha Xing

**Affiliations:** 1 Chengdu Women’s and Children’s Central Hospital, School of Medicine, University of Electronic Science and Technology of China, Chengdu, 611731 China; 2 Hongqiao International Institute of Medicine, Tongren Hospital, Shanghai Jiao Tong University School of Medicine, Shanghai, 200336 China

**Keywords:** Atherosclerosis, Untargeted lipidomics, *ApoE*^*−/−*^ mice, Phosphocholines, Lysophosphocholines

## Abstract

**Objective:**

Atherosclerosis (AS) is the major cause of cardiovascular disease, and dyslipidemia is a principal determinant of the initiation and progression of AS. Numerous works have analyzed the lipid signature of blood, but scarce information on the lipidome of vascular tissue is available. This study investigated the lipid profile in the aorta of *ApoE*^*−/−*^ mice.

**Method:**

*ApoE*^*−/−*^ mice were randomly divided into two groups: (1) the normal diet (ND) group and (2) the high-fat diet (HFD) group. After feeding for 8 weeks, the plasma low-density lipoprotein (LDL), total cholesterol (TC), and triglyceride (TGs) levels were measured. UHPLC-Q Exactive plus MS was used to assess the lipid profile using both positive and negative ionization modes.

**Results:**

LDL and TC levels were significantly increased in HFD mice, and lipid deposition, plaque area and collagen fiber levels were increased in HFD group. In addition, a total of 131 differential lipids were characterized, including 57 lipids with levels that were increased in the HFD group and 74 with levels that were decreased. Further analysis revealed that the levels of several differentially expressed phosphocholines (PCs) and lysophosphocholines (LPCs) were significantly increased. These PCs included PC (38:3), PC (36:4), PC (36:3), PC (36:2), PC (36:1), PC (34:1e), PC (34:1), PC (32:1), PC (18:0/18:1), and PC (38:5), and the LPCs included LPC (18:1), LPC (18:0) and LPC (16:0).

**Conclusion:**

Our findings indicate the presence of a comprehensive lipid profile in the vascular tissue of atherosclerotic mice, particularly involving PC and LPC, which exhibited significantly increased levels in AS.

**Supplementary Information:**

The online version contains supplementary material available at 10.1186/s12986-022-00723-y.

## Background

Cardiovascular and cerebrovascular diseases caused by atherosclerosis (AS) remain the leading cause of morbidity and mortality worldwide [1, 2]. AS is characterized by lipid disturbance, which leads to the formation of fibrofatty lesions in the arterial wall, subsequent hardening and narrowing of the arteries, and eventually the formation of plaques in the vessel wall [3, 4]. Lipid disturbance, vascular dysfunction, smooth muscle cell proliferation, oxidative stress and inflammation exacerbate the progression of atherosclerosis [5]. A large number of studies show that lipid disorder is a principal determinant of the initiation and progression of AS [3]. Endogenous lipids are the major constituents of cell membranes, which are extensively involved in the structural compartmentalization of signal transduction. Moreover, these lipids act as potential pathophysiological mediators and participate in regulating several intracellular functions, such as proliferation, apoptosis, oxidative stress and inflammation [6]. Atherosclerosis is closely linked to lipid overload, including increased plasma levels of triglycerides (TG), low-density lipoprotein (LDL), intermediate-density lipoprotein (IDL) and VLDL [7, 8]. For example, LDL, a particle that carries cholesterol through the blood, is not only the initiator but also an accelerator of AS [9, 10]. Lipoprotein(a), a low-density lipoprotein (LDL) cholesterol-like particle that binds to apolipoprotein (A), has a potential causal relationship with atherosclerotic cardiovascular disease [11].

Lipidomics is a powerful analytical tool for analyzing the lipid composition in biological samples under physiological and pathological conditions to elucidate disease pathogenesis, identify biomarkers or study the effect and mechanism of drugs [12, 13]. Liquid chromatography coupled to MS (LC‒MS) is undoubtedly considered the current gold standard for the quantification of lipids [14]. Lipidomics has been widely used in screening and mechanistic research on lipid biomarkers in atherosclerotic diseases in recent years. For example, the plasma lipid levels in subjects with TG/HDL-C levels ≥ 3.287 were found to be significantly different from those in subjects with TG/HDL-C levels < 1.391. The downregulated lipid including sphingomyelins (SM) (36:2), SM (38:2), SM (40:2), SM (41:2), SM (41:1), SM (42:4), SM (42:3), SM (42:1), electrophoresis (CE) (16:0), CE (18:0), CE (18:1), PC (38:2), and PC (36:2e)/PC (36:1p), and PE (38:1), to the contrary, all TG species and phosphatidylserine (PS) (36:1) were increased [15]. In patients with large artery atherosclerotic cerebrovascular disease, 276 sphingolipids were detected in the plasma via lipidomic analysis, including SM (n = 162), glycosphingolipids (n = 72), Cer (n = 39), and ceramide phosphates (n = 3) [16]. Moreover, Wang et al. [17] identified nine types of 732 lipid components in the plasma of *ApoE*^*−/−*^ mice, including 11 lysophosphatidylethanolamine (LPE), 12 SM, 15 LPC, 27 cholesteryl esters (CE), 35 ceramides (Cer), 38 diacylglycerol (DAG), 60 phosphatidylethanolamine (PE), 64 PC, and 470 triglyceride (TAG). Therefore, further comprehensive exploration of the lipid characteristics related to atherosclerosis, including in serum and vascular tissue, is conducive to enabling an in-depth understanding of the pathogenesis of atherosclerosis and is also expected to provide new insights for the diagnosis, prevention and cure of atherosclerosis.

In summary, omics approaches are used to identify lipid profiles in plasma, serum, and plaques to establish a disease prediction model, elucidate disease pathogenesis, improve diagnostic efficiency and provide drug targets. To date, due to the limited sample size, although Jihan Talib et al*.* have developed a new Multi-ABLE method that can be used to assess small tissue samples (< 5 mg) and have performed aortic lipidomics in *ApoE*^*−/−*^ mice [18], there has been no literature describing the use of classic LC‒MS techniques to assess the profile of vascular lipids in *ApoE*^*−/−*^ mice. Therefore, we used classic LC‒MS/MS lipidomics technology to provide a basis for elucidating the lipid profile of atherosclerotic thoracic aortas in *ApoE*^*−/−*^ mice.

## Materials and methods

### Reagents and materials

The chemicals methanol (No. A452-4), acetonitrile (No. A998-4), formic acid (No. A117-50), and isopropanol (No. A451-4) were purchased from ThermoFisher (ThermoFisher, Waltham, MA, USA), ammonium acetate (10001218) was purchased from Sinopharm (Sinopharm, Beijing, China), Lyso PC17:0 (No. 855676P) was purchased from Avanti (Avanti, Alabama, USA), chloroform (No. G75915B) was purchased from Greagent (Greagent, Shanghai). Distilled water was purchased from Watsons (Watsons, Guangzhou, China). All aqueous solutions were prepared using purified water at a Milli-Q grade.

### Animal model establishment and specimen acquisition

This study was approved by the Chengdu Women’s and Children’s Central Hospital Ethics Committee. *ApoE*^*−/−*^ mice (4 weeks old, male) were purchased from Jiangsu Jicui Yaokang Biotechnology Co., Ltd. Animals were kept in a specific pathogen-free (SPF) environment. The feeding conditions included a light–dark cycle for 12 h, temperature 22–25 °C, and a relative humidity of 60%. The mice were divided into two groups: (1) mice were fed a normal diet, and (2) mice were fed a high-fat diet (Yangzhou Promoter Biotechnology Co., Ltd., D12108C). After feeding for 8 weeks, the mice were fasted overnight and anesthetized with sodium pentobarbital solution (1%, 10 mL/kg) by intraperitoneal injection. The plasma was separated from the blood, which was collected from the abdominal aorta, after standing for 2 h and centrifugation at 8000 rpm for 8 min. The plasma was transferred to an EP tube and preserved at − 80 °C refrigerator before being used for analysis. The thoracic aorta was peeled off and preserved at − 80 °C refrigerator.

### The measurement of serum TC, TG and LDL levels

The cryopreserved plasma (100 μL) obtained following fasting was thawed and then centrifuged. The biochemical markers associated with lipid metabolism, including the levels of LDL, TC, and TG, were detected using a commercial kit according to the manufacturer’s instructions (Changchun Huili Biotech Co., Ltd.).

### Histopathologic analysis

The heart, including the aortic root, was fixed in paraformaldehyde. Then, the tissues were embedded in OCT compound and sliced into sections (7 μm) [19]. Then, HE and Oil Red O staining were performed to observe the plaque area and lipid deposition, respectively. Furthermore, Masson’s trichrome staining was performed to observe the collagen area.

All images were photographed using a panoramic MIDI scanning microscope (3DHISTECH, Hungary) and then quantified using Image-Pro Plus 6.0 software. The plaque area was measured as the total area of plaque. Lipid deposition is presented as the percentage of positive cells in total plaque. The collagen area was measured as the collagen-positive area.

### Immunostaining

As previously described, the expression of CD68 in the aortic root was assessed by immunostaining [20]. Briefly, the frozen sections were permeabilized with Triton X-100 (0.2%, 15 min) at room temperature. After incubation with rabbit anti-CD68 (1:100, Servicebio) at 4 °C overnight, the sections were washed, a secondary antibody was added, and the samples were incubated. Finally, DAPI was added to stain the nuclei. Images were acquired using a panoramic MIDI scanning microscope (3DHISTECH, Hungary) and then quantified using Image-Pro Plus 6.0 software.

### Lipid extraction

Due to the limitation of sample weight, the thoracic aorta vessels of every two mice were combined in the analysis. After quickly freezing with liquid nitrogen, the samples were placed in and stored at − 80 °C refrigerator until analysis. Lipids were extracted as follows: (1) 20 μL Lyso PC-17:0 (0.1 mg/mL, dissolved in methanol) was added to the samples as internal standard to reflect the repeatability of the extraction. And then mixed the samples with methanol: water (1/1, vol/vol, 300 μL). Subsequently, the samples were subjected to grinding with two small steel balls (60 Hz, 2 min) in grinder (Tissuelyser-48, Shanghai) and then added 300 μL chloroform for ultrasonication for 10 min, and stored at − 20 °C for 20 min. After centrifugation for 10 min, 200 μL of the chloroform layer was transferred into a new centrifuge tube. (2) The residual samples were added to chloroform:methanol (2/1, vol/vol) and then vortexed for 30 s. The samples were ultrasonicated for 10 min, placed at − 20 °C for 20 min, and centrifuged for 10 min, and then taken 150 μL of the chloroform layer into the centrifuge tube. (3) The two chloroform layers were combined and then dried under a nitrogen stream. The samples were re-dissolved in 400 μL isopropanol:methanol (1/1, vol/vol), vortexed for 30 s and sonicated for 3 min. Before detection, the solution was filtered through a 0.22 μm organic phase pinhole filter. All sonication was performed in an ice-water bath, and centrifugation was performed at 13,000 rpm at 4 °C. QC samples were prepared by mixing aliquots of all samples, and then were injected at regular intervals (every 6 samples) into the samples throughout the analysis procedure to evaluate the stabilityof the equipment. All chemicals and solvents were analytical or HPLC grade.

### Lipidomic profiling using LC‒MS

Lipid profiling was performed using an UPLC system (Waters, Japan) coupled to a Q-Exactive plus mass spectrometer equipped with a heated electrospray ionization (ESI) source (Thermo Fisher Scientific, Waltham, MA, USA). In detail, the samples were separated using an ACQUITY UPLC BEH C8 column (100 mm × 2.1 mm, 1.7 um, Waters) and detected using ESI positive and ESI negative ion modes. During the analysis, the samples were kept at 4 °C. The gradient elution system consisted of two mobile phases, (A) acetonitrile: water (6:4, v:v, containing 10 mmol ammonium formate, 0.1% formic acid) and (B) isopropanol: acetonitrile (9:1, v:v, containing 10 mmol ammonium formate, 0.1% formic acid). The flow rate was 0.26 mL/min, and the column temperature was 55 °C. The injection volumes were 2 μL and 4 μL in positive and negative modes, respectively. The separation was achieved using the following gradient: 0 min, 32% B; 1.5 min, 32% B; 15.5 min, 85% B; 15.6 min, 97% B; 18.0 min, 97% B; 18.1 min, 32% B; 20.0 min, 32% B.

The mass spectrometer was operated in both positive electrospray ionization (ESI+) mode and negative electrospray ionization (ESI−) mode. The acquisition parameters: the spray voltage value of positive ions is 3.5 kv, and 3.0 kv negative ions; Capillary Temp 300 °C; Mass range (m/z): 150–1500; Full ms resolution: 70,000; MS/MS resolution: 17,500.

### IPA analysis

Ingenuity pathway analysis (IPA) is a widely used bioinformatics software for predicting the correlations among components, targets, pathways, and diseases [21, 22]. The HMDB number of differential lipids was downloaded from the HMDB database (https://hmdb.ca/) [23]. In canonical pathway analysis and analyses of disease and function, we took − log (*P* value) > 2 as the threshold and then defined Z score > 2 and Z score < − 2 as the threshold of significant activation and inhibition, respectively. The consistency scores were calculated by assessing regulatory effects and molecular networks, and the higher the consistency score was, the more accurate the regulatory effect analysis results. In addition, we used a *P* value of overlap < 0.05 as the inclusion criteria for upstream regulators.

### Statistical analysis

The original data obtained from Q Exactive plus (LC‒MS/MS) analysis were processed by Lipid Search software. The molecular structure of lipids and the additive mode of its positive and negative ions were confirmed according to the parent ions and multistage mass spectrometry data of each individual sample. To sort the original data matrix, results were aligned according to a certain retention time range. The peak signals in each sample were normalized, by converting its signal intensity into the relative intensity in the spectrum, and multiplying it by 100,000. Lipids which the relative standard deviation (RSD) of QC greater than 30% were discarded. These highly variable ions should be rejected as unacceptable for the analysis. Following the extraction of data, peaks with missing values (ion intensity = 0) in more than 50% of groups were removed and replaced the zero value with half of the minimum value. Positive and negative ion data were combined into a data matrix. Principal component analysis (PCA) was carried out to monitor the overall sample distribution and the stability of the whole analysis process. Orthogonal partial least squares-discriminant analysis (OPLS-DA) and partial least squares-discriminant analysis (PLS-DA) were used to authenticate the results regarding the differential lipids. Variable importance of projection (VIP) value was used to assess the variable importance. In addition, the Kyoto Encyclopedia of Genes and Genomes (KEGG) pathway database was used to analyze metabolic pathway enrichment. The statistical graphs were generated by GraphPad Prism 8 software, and a two-tailed Student’s T test was used for statistical analysis. All data are presented as the means ± standard errors of the means, SEM). Differential lipids were selected with VIP values > 1.0 and *p* values < 0.05.

## Results

### Effects of a high-fat diet on the lipid profile in ***ApoE***^***−/−***^ mice

**Fig. 1:**
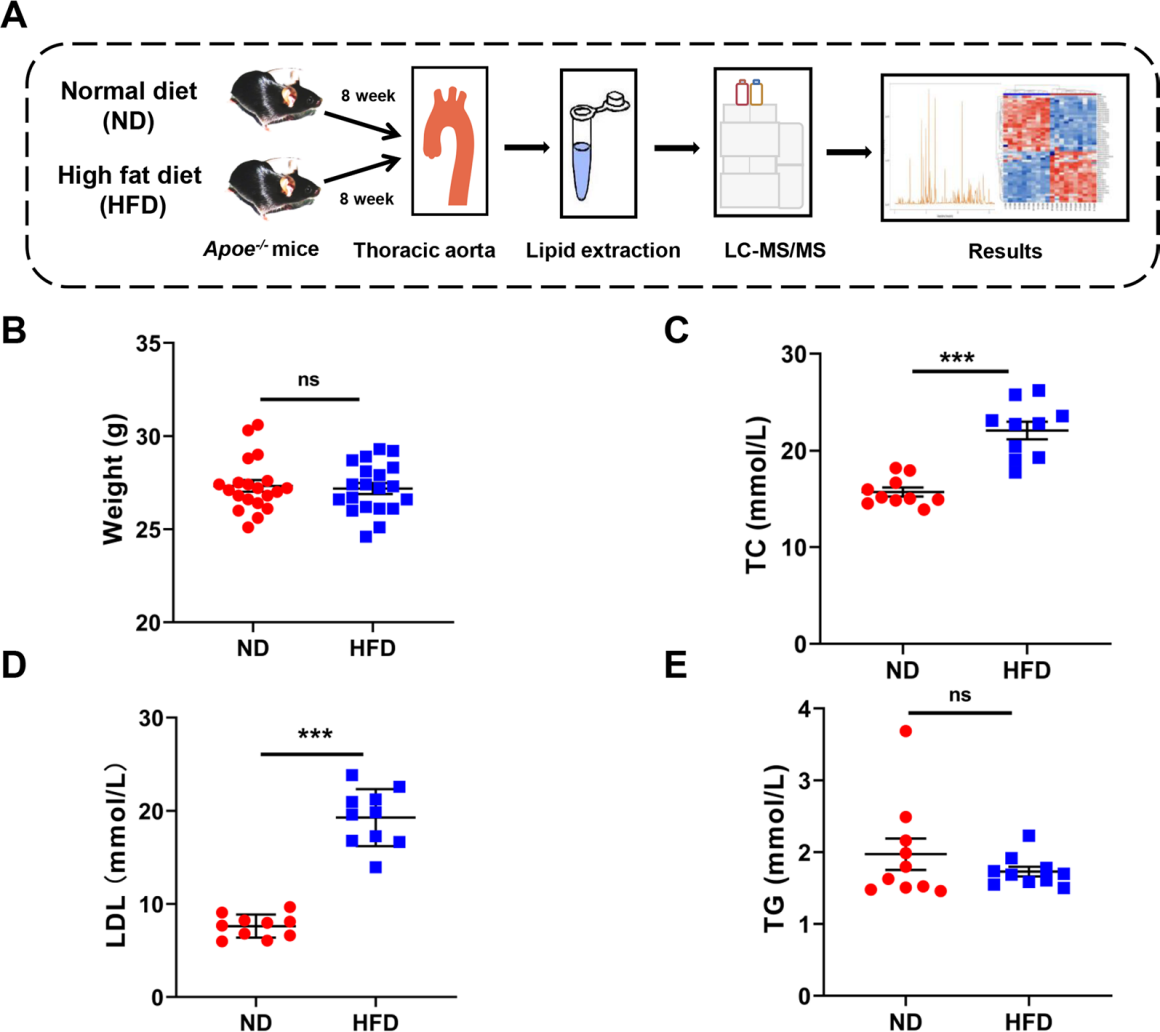
Study design of lipidomics research and the successful establishment of the atherosclerosis model. **A** The blood vessels of mice were collected, and lipids were analyzed by LC/MS. **B** The difference in body weight between ND- and HFD-fed *ApoE*^*−/−*^ mice (n = 20 per group). **C** Measurement of TC content in plasma (n = 10 per group). **D** Measurement of LDL content in plasma (n = 10 per group). **E** Measurement of TG content in the plasma of mice (n = 10 per group). All data are presented as the means ± SEMs. ***P < 0.001 versus the ND group. ns, not statistically significant. LC/MS, liquid chromatography‒mass spectrometry; TC, total cholesterol; LDL, low-density lipoprotein; TG, triglyceride; ND, normal diet; HFD, high-fat diet

We used male *ApoE*^*−/−*^ mice fed a high-fat diet to establish an animal model of atherosclerosis and then collected blood vessel tissue to extract lipids for mass spectrometry analysis. The study design is shown in Fig. 1A. In brief, *ApoE*^*−/−*^ mice were fed a high-fat diet (HFD) for 8 weeks to establish an atherosclerosis model, while *ApoE*^*−/−*^ mice fed a normal diet (ND) were used as a control. The lipid species in thoracic aorta were assessed via HPLC‒MS/MS. The body weight was not significantly different between the ND group and the HFD group (Fig. 1B). The plasma levels of TC, LDL-C, and TG were detected to investigate the effects of HFD on total lipids. The levels of TC and LDL-C in the HFD group were significantly higher than those in the ND group, while no significant differences in TG levels were observed; Fig. 1C–E illustrates the details.

### Assessment of atherosclerotic plaque formation in ***ApoE***^***−/−***^ mice

**Fig. 2:**
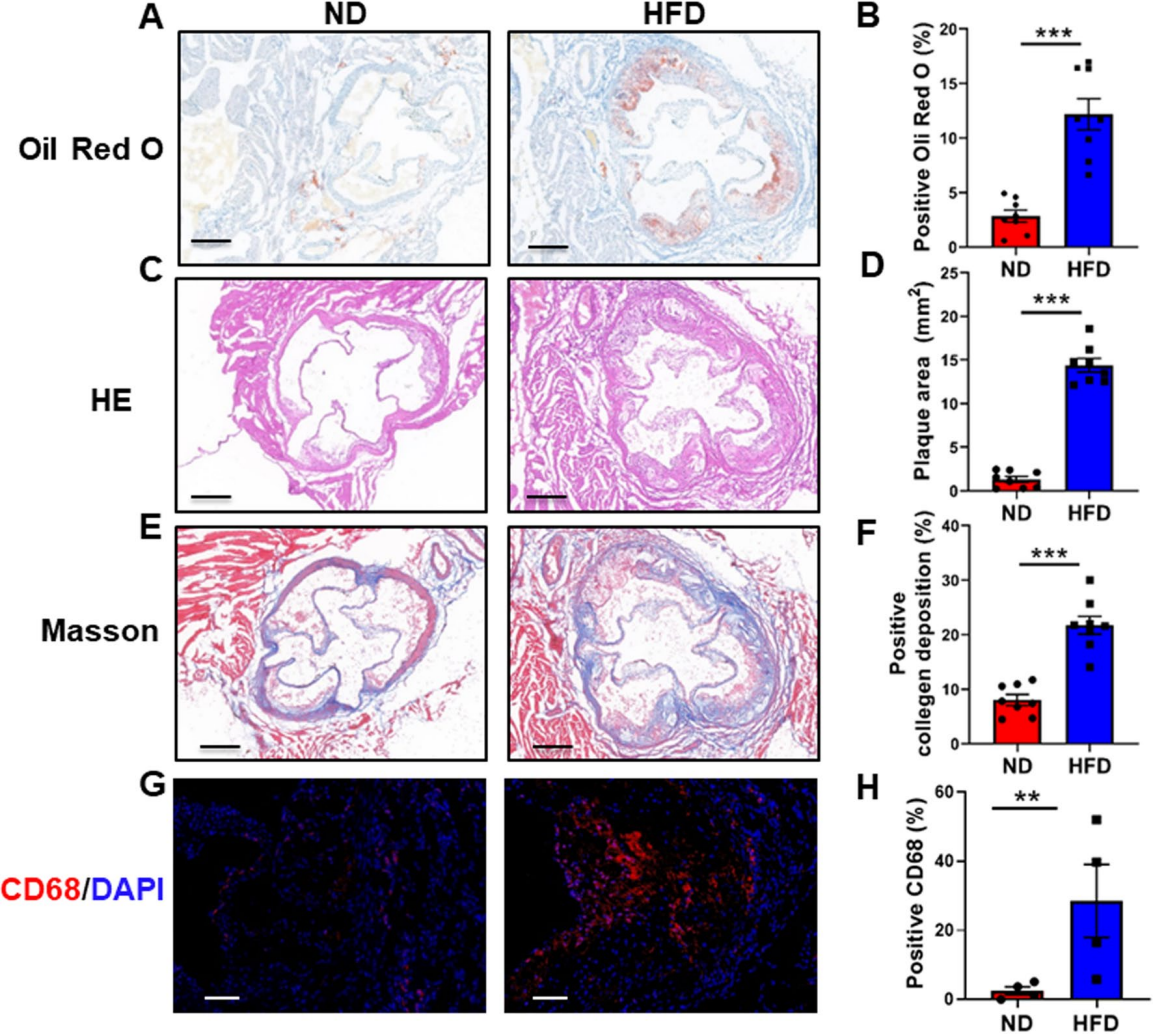
Assessment of atherosclerotic plaque formation in *ApoE*^*−/−*^ mice. **A** Representative photographs of HE staining show increased plaque formation in the HFD group. Magnified ×50. **B** Quantification data of HE staining (n = 8 per group). **C** Representative photographs of Oil Red O staining show increased lipid deposition in the HFD group. Magnified ×50. **D** Quantification data of Oil Red O staining (n = 8 per group). **E** Representative photographs of Masson’s trichrome staining show increased collagen content in the HFD group. Magnified ×50. **F** Quantification data of Masson’s trichrome staining (n = 8 per group). **G** Representative photographs of immunofluorescence staining of CD68 show increased macrophage in the HFD group. Magnified ×200. **H** Quantification data of CD68 expression (n = 4 per group). All data are presented as the means ± SEMs. ***P < 0.001 versus the ND group. HE, hematoxylin–eosin; ND, normal diet; HFD, high-fat diet

To verify that the model of atherosclerotic mice was successful, HE and Oil Red O staining were performed in frozen sections of the aortic root of *ApoE*^*−/−*^ mice. The results of HE staining showed that 8 weeks of HFD treatment significantly increased plaque formation (Fig. 2A, B). Oil Red O staining showed that lipid deposition was also significantly increased in HFD-treated *ApoE*^*−/−*^ mice (Fig. 2C, D). Masson's trichrome staining showed that the collagen content was significantly increased in HFD-treated *ApoE*^*−/−*^ mice (Fig. 2E, F). In addition, the immunofluorescence staining of CD68 expression revealed that free monocyte/macrophage levels in the aortic root were increased (Fig. 2G, H). The above results revealed that *ApoE*^*−/−*^ mice treated with HFD did indeed undergo the pathological changes associated with atherosclerosis, which suggested the success of animal model establishment.

### UHPLC-Q Exactive MS-based lipidomics analysis

UHPLC-Q Exactive Plus-MS was used to lipidomic analysis of the thoracic aorta samples. The molecular structure of lipids and the additive mode of the positive and negative ions were identified according to the parent ions and multistage mass spectrometry data obtained from each individual sample. The base peak chromatograms (BPCs) of positive ions and negative ions in the ND group and HFD group are shown in Additional file 1: Fig. S1, respectively.

**Fig. 3:**
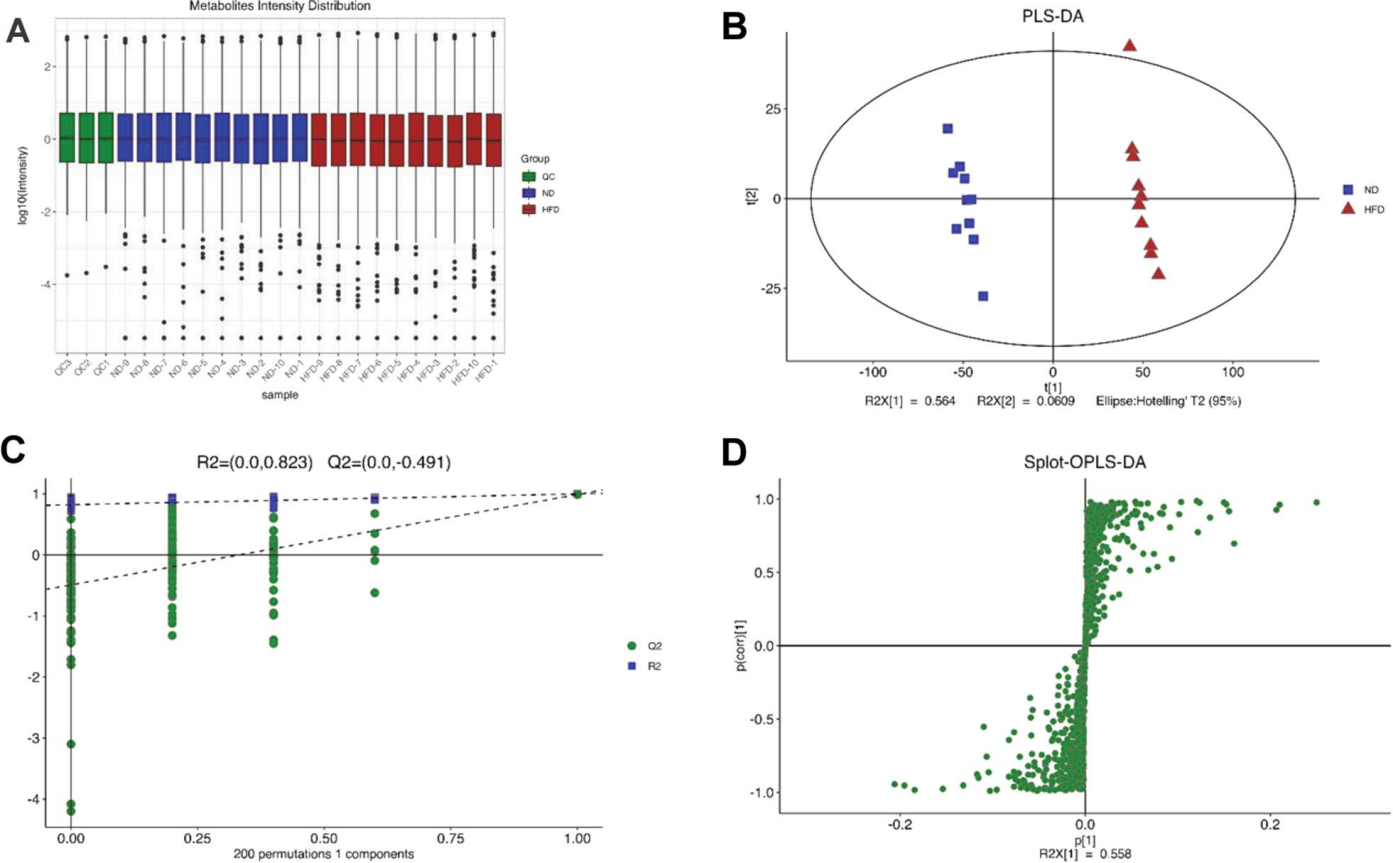
UHPLC-Q Exactive MS-based lipidomics analysis. **A** Lipid intensity distribution of the QC samples. The Y coordinate is the log10 value of the mass spectrum intensity. **B** PLS-DA analysis performed the optimization separated between ND and HFD groups. **C** Permutation analysis. R2x (cum) and R2y (cum) represent the cumulative interpretation rate of the model in the x-axis and y-axis directions, respectively. Cum represents the accumulation of several principal components. Q2 (cum) represents the cumulative prediction rate of the model. The value (green) of all Q2 on the left is lower than the initial value on the right, which shows that the model is effective. **D** Splot analysis of OPLS-DA. The characteristic value of the influence of lipids on the comparison group was taken as the abscissa of the Splot, while the correlation between the sample score and lipids was taken as the ordinate. PLS-DA, partial least squares discrimination analysis; OPLS-DA, orthogonal partial least squares discriminant analysis; ND, normal diet; HFD, high-fat diet

To evaluate the stability of the equipment, the lipid intensity distribution of the QC samples is shown in Fig. 3A, where the Y coordinate is the log10 value of the mass spectrum intensity. PCA was used first to monitor the overall distribution of the samples and the stability of the entire analysis process, as shown in (Additional file 2: Fig. S2). The distance between the two coordinate points on the score chart was far on the score map, indicating that there was a significant difference between the two samples. PLS-DA and OPLS-DA were used to identify differential lipids between groups (Fig. 3B, Additional file 2: Fig. S2). A random ranking method, the response ranking test, was used to evaluate the accuracy of the OPLS model. The value (green) of all Q2 on the left was lower than the initial value on the right, which shows that the model was effective (Fig. 3C). An S-plot showed that all the points were distributed in the first and third quadrants, and the closer the lipid molecules were to the lower left or upper right of the plot, the more significant the differences were (Fig. 3D).

### Analysis of lipidomic profiling and screening of differential lipids

**Fig. 4:**
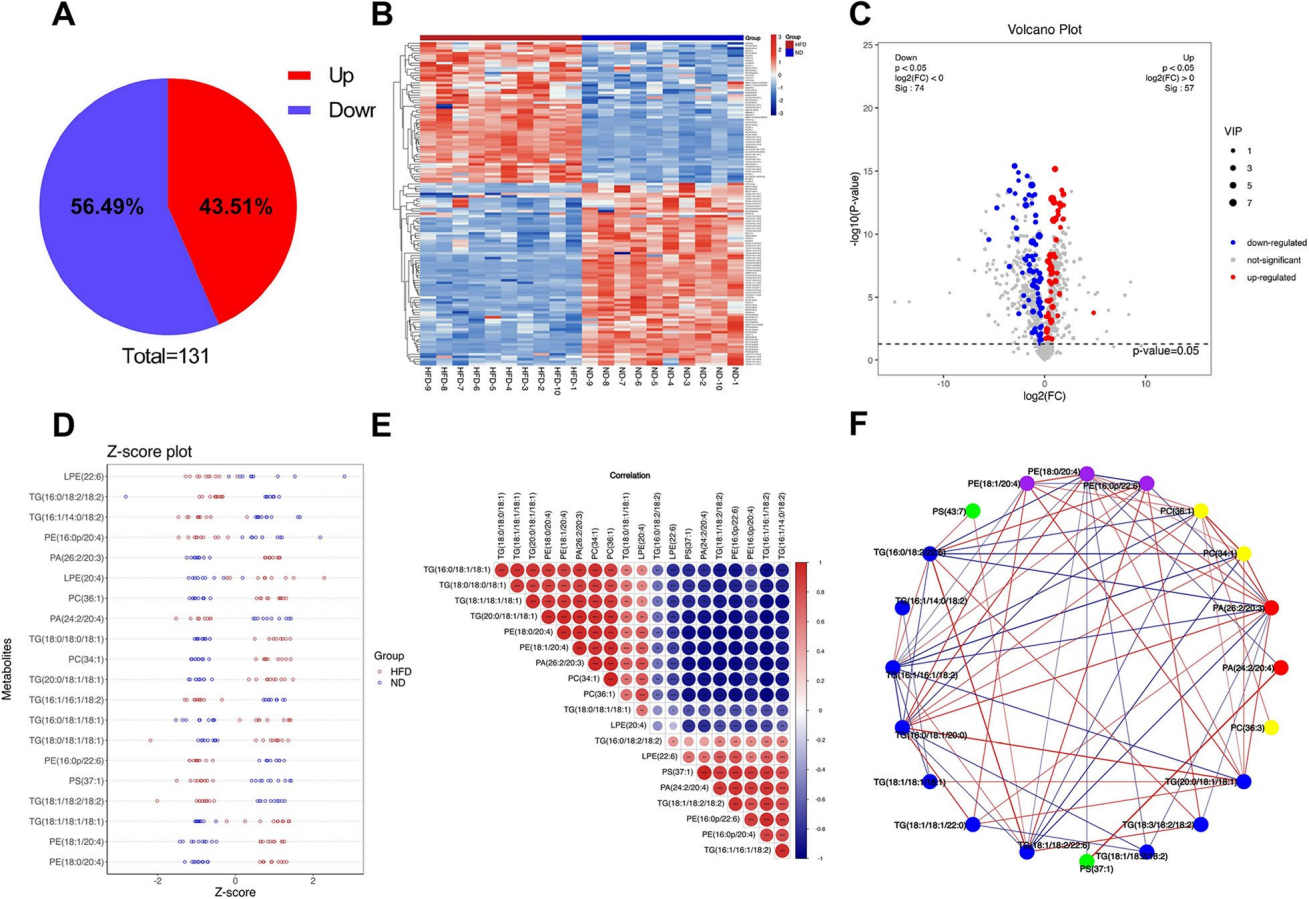
Analysis of lipidomic profiling and identification of differential lipids. **A** Differentially expressed lipids were identified (VIP > 1, *P* < 0.05). **B** Heatmap of the levels differentially expressed lipids. Increased levels are indicated in red, and decreased levels are indicated in blue. **C** Volcano plots of the levels of differentially expressed lipids. Red indicates increased levels, blue indicates decreased levels, and gray indicates no statistical significance. **D** Z score analysis of the top 20 lipids with large differences ranked by VIP. **E** Correlation analysis of the top 20 lipids. Red indicates a positive correlation, blue indicates a negative correlation. The larger the dot is, the larger the correlation coefficient between the two variables. **F** The correlation network diagram of the top 20 lipids (*P* < 0.05, correlation > 0.95). All data are presented as the means ± SEMs. *P < 0.05, **P < 0.01 and ***P < 0.001 versus the ND group. TG, triglyceride; PE, phosphatidylethanolamine; PC, phosphatidylcholine; PS, phosphatidylserine; PA, phosphatidic acid; SM, sphingomyelin; LPE, lysophosphatidylethanolamine; VIP, variable important in projection; ND, normal diet; HFD, high-fat diet

Based on the PLS-DA model, a total of 131 differential lipids were identified according to the conditions of VIP > 1 and *P* < 0.05. A total of 57 lipids exhibited increased levels, and 74 exhibited decreased levels in the HFD group (Fig. 4A). The details of the differential lipids are shown in Table 1. As shown in Fig. 4B, C, the expression levels of the 131 differential lipids were used to draw a heatmap and volcano plots for comparisons between the HFD and ND groups. Unlike in the ND group, the top 20 lipid components included PE (18:0/20:4), PE (18:1/20:4), TG (18:1/18:1/18:1), TG (18:1/18:2/18:2), PS (37:1), PE (16:0p/22:6), TG (18:0/18:1/18:1), TG (16:0/18:1/18:1), TG (16:1/16:1/18:2), TG (20:0/18:1/18:1), PC (34:1), TG (18:0/18:0/18:1), PA (24:2/20:4), PC (36:1), LPE (20:4), PA (26:2/20:3), PE (16:0p/20:4), TG (16:1/14:0/18:2), TG (16:0/18:2/18:2), and LPE (22:6). The details of the differential lipids that were analyzed in the top 20 significantly different metabolites sorted by VIP are shown in Table 2. We performed Z score analysis on the top 20 significantly different lipids sorted by VIP and transformed the data of different magnitudes into a unified measure of Z score for comparison (Fig. 4D). To study the degree of correlation between lipid levels, we performed correlation analysis using the top 20 significantly different lipids sorted by VIP and used the Pearson correlation coefficient to measure the degree of linear correlation between lipid levels. A positive correlation is indicated in red, while a negative correlation is indicated in blue. The larger the dot is, the larger the correlation coefficient between the two variables (Fig. 4E). In addition, we selected the relationship pair with *p* value < 0.05 and correlation > 0.95 to draw a correlation network diagram (Fig. 4F).

**Table 1:** Differential lipids identified in the thoracic aorta of HFD versus ND fed *apoE*^*−/−*^ mice

ID	Lipid	Class	Formula	VIP	*P*-value
89	PE (18:0/20:4)	PE	C_43_ H_76_ O_8_ N_1_ P_1_	8.06556	1.6E−13
91	PE (18:1/20:4)	PE	C_43_ H_74_ O_8_ N_1_ P_1_	6.77589	7.8E−12
53	TG (18:1/18:1/18:1)	TG	C_57_ H_104_ O_6_	6.6631	4.9E−09
55	TG (18:1/18:2/18:2)	TG	C_57_ H_100_ O_6_	6.6448	4E−10
1232	PS (37:1)	PS	C_43_ H_80_ O_10_ N_1_ P_1_	6.3032	1.3E−10
93	PE (16:0p/22:6)	PE	C_43_ H_72_ O_7_ N_1_ P_1_	5.94693	1.3E−14
52	TG (18:0/18:1/18:1)	TG	C_57_ H_106_ O_6_	5.18991	0.0007
46	TG (16:0/18:1/18:1)	TG	C_55_ H_102_ O_6_	5.0121	1.4E−08
42	TG (16:1/16:1/18:2)	TG	C_53_ H_94_ O_6_	4.9633	5.1E−13
62	TG (20:0/18:1/18:1)	TG	C_59_ H_110_ O_6_	4.91587	6.3E−12
956	PC (34:1)	PC	C_42_ H_82_ O_8_ N_1_ P_1_	4.8359	3E−13
51	TG (18:0/18:0/18:1)	TG	C_57_ H_108_ O_6_	4.34905	5.3E−07
615	PA (24:2/20:4)	PA	C_47_ H_79_ O_8_ P_1_	4.25154	1.5E−10
963	PC (36:1)	PC	C_44_ H_86_ O_8_ N_1_ P_1_	3.98438	3.9E−13
85	LPE (20:4)	LPE	C_25_ H_42_ O_7_ N_1_ P_1_	3.92548	5.8E−05
704	PA (26:2/20:3)	PA	C_49_ H_85_ O_8_ P_1_	3.88784	6.9E−16
87	PE (16:0p/20:4)	PE	C_41_ H_72_ O_7_ N_1_ P_1_	3.77053	3.3E−07
165	TG (16:1/14:0/18:2)	TG	C_51_ H_92_ O_6_	3.72125	7.4E−08
132	TG (16:0/18:2/18:2)	TG	C_55_ H_98_ O_6_	3.54014	0.01038
115	LPE (22:6)	LPE	C_27_ H_42_ O_7_ N_1_ P_1_	3.43674	0.00013
1319	PEt (46:10)	PEt	C_51_ H_79_ O_8_ P_1_	3.36612	1.1E−06
61	TG (18:0/18:1/20:0)	TG	C_59_ H_112_ O_6_	3.34356	6.7E−14
136	TG (16:0/18:2/22:6)	TG	C_59_ H_98_ O_6_	3.3203	4E−16
86	PE (16:0/20:4)	PE	C_41_ H_72_ O_8_ N_1_ P_1_	3.27435	5.9E−08
170	TG (18:1/18:2/22:6)	TG	C_61_ H_100_ O_6_	3.08254	3.5E−14
601	SM (d17:1/16:0) + CH3COO	SM	C_40_ H_80_ O_8_ N_2_ P_1_	3.01966	0.00542
964	PC (36:2)	PC	C_44_ H_84_ O_8_ N_1_ P_1_	2.95564	1.9E−05
69	TG (18:1/18:1/22:0)	TG	C_61_ H_114_ O_6_	2.73606	2.8E−10
965	PC (36:3)	PC	C_44_ H_82_ O_8_ N_1_ P_1_	2.69664	7.3E−08
146	PE (18:0p/22:6)	PE	C_45_ H_76_ O_7_ N_1_ P_1_	2.66488	1.3E−07
38	TG (16:1/14:0/18:1)	TG	C_51_ H_94_ O_6_	2.6602	0.00248
1011	PS (41:4)	PS	C_47_ H_82_ O_10_ N_1_ P_1_	2.60725	3.6E−08
45	TG (18:0/16:0/18:1)	TG	C_55_ H_104_ O_6_	2.5166	0.01558
118	PS (18:0/22:6)	PS	C_46_ H_76_ O_10_ N_1_ P_1_	2.4887	1.5E−06
57	TG (18:3/18:2/18:2)	TG	C_57_ H_96_ O_6_	2.48633	3.2E−11
8	DG (18:1/18:2)	DG	C_39_ H_70_ O_5_	2.47904	0.0062
64	TG (20:1/18:1/18:2)	TG	C_59_ H_106_ O_6_	2.4762	9.7E−08
145	TG (16:1/18:2/18:3)	TG	C_55_ H_94_ O_6_	2.42793	3.7E−08
41	TG (16:0/16:0/18:1)	TG	C_53_ H_100_ O_6_	2.3946	0.00317
92	PE (18:1p/20:4)	PE	C_43_ H_74_ O_7_ N_1_ P_1_	2.31574	5.8E−07
1097	PS (43:7)	PS	C_49_ H_80_ O_10_ N_1_ P_1_	2.31528	5.8E−09
966	PC (36:4)	PC	C_44_ H_80_ O_8_ N_1_ P_1_	2.29038	5.3E−07
126	TG (16:1/12:0/18:1)	TG	C_49_ H_90_ O_6_	2.28252	0.00013
158	PE (18:1p/22:6)	PE	C_45_ H_74_ O_7_ N_1_ P_1_	2.24968	3.2E−12
7	DG (18:1/18:1)	DG	C_39_ H_72_ O_5_	2.19876	0.01991
230	PE (18:0/22:5)	PE	C_45_ H_78_ O_8_ N_1_ P_1_	2.17785	1.4E−12
141	PE (16:1/22:6)	PE	C_43_ H_70_ O_8_ N_1_ P_1_	2.16375	2.5E−08
50	TG (18:1/17:1/18:2)	TG	C_56_ H_100_ O_6_	2.11837	5.5E−09
65	TG (16:0/18:1/22:6)	TG	C_59_ H_100_ O_6_	2.11259	6.6E−10
994	SM (d34:1)	SM	C_39_ H_79_ O_6_ N_2_ P_1_	2.10728	1.4E−07
137	TG (20:5/18:2/18:2)	TG	C_59_ H_96_ O_6_	2.02499	8.4E−13
654	So (d18:0)	So	C_18_ H_39_ O_2_ N_1_	2.02244	0.00334
671	PA (26:4/20:4)	PA	C_49_ H_79_ O_8_ P_1_	1.98625	5.1E−07
1088	PS (37:2)	PS	C_43_ H_78_ O_10_ N_1_ P_1_	1.98572	1.3E−05
116	PE (18:0/18:2)	PE	C_41_ H_76_ O_8_ N_1_ P_1_	1.96199	1.9E−05
212	PE (18:0p/22:4)	PE	C_45_ H_80_ O_7_ N_1_ P_1_	1.93691	5.9E−06
67	TG (16:0/18:1/24:0)	TG	C_61_ H_116_ O_6_	1.93571	6.2E−12
39	TG (16:1/14:1/18:2)	TG	C_51_ H_90_ O_6_	1.91479	2.4E−10
63	TG (20:1/18:1/18:1)	TG	C_59_ H_108_ O_6_	1.89866	0.02543
662	LPE (16:0p)	LPE	C_21_ H_42_ O_6_ N_1_ P_1_	1.88264	0.00349
68	TG (18:2/18:2/22:6)	TG	C_61_ H_98_ O_6_	1.87898	2.7E−10
9	DG (18:2/18:2)	DG	C_39_ H_68_ O_5_	1.85778	0.0002
131	TG (15:0/18:1/18:2)	TG	C_54_ H_98_ O_6_	1.85026	1.1E−11
94	PE (20:4/20:4)	PE	C_45_ H_72_ O_8_ N_1_ P_1_	1.83114	4.4E−07
90	PE (16:0p/22:4)	PE	C_43_ H_76_ O_7_ N_1_ P_1_	1.82058	2.3E−08
441	TG (20:2/18:2/18:2)	TG	C_59_ H_102_ O_6_	1.81929	7.7E−14
220	PE (16:0p/18:2)	PE	C_39_ H_72_ O_7_ N_1_ P_1_	1.79582	7.2E−14
73	TG (18:1/18:1/24:0)	TG	C_63_ H_118_ O_6_	1.78091	2.9E−12
1336	SM (d41:3) + CH3COO	SM	C_48_ H_92_ O_8_ N_2_ P_1_	1.77416	3E−05
595	CL (18:2/18:1/18:2/18:2)	CL	C_81_ H_142_ O_17_ P_2_	1.7722	1.1E−07
161	TG (14:0/18:2/18:3)	TG	C_53_ H_92_ O_6_	1.76528	7.3E−06
974	PC (38:5)	PC	C_46_ H_82_ O_8_ N_1_ P_1_	1.74598	6.7E−09
594	CL (18:2/18:1/18:1/18:2)	CL	C_81_ H_144_ O_17_ P_2_	1.74508	3.2E−14
630	PA (26:3/22:6)	PA	C_51_ H_81_ O_8_ P_1_	1.67472	0.00105
336	PE (16:0p/20:5)	PE	C_41_ H_70_ O_7_ N_1_ P_1_	1.67088	2.5E−15
575	LPC (18:1)	LPC	C_26_ H_52_ O_7_ N_1_ P_1_	1.62655	1.2E−07
60	TG (19:1/18:1/18:1)	TG	C_58_ H_106_ O_6_	1.62541	1.8E−06
206	TG (15:0/18:2/18:2)	TG	C_54_ H_96_ O_6_	1.59933	1.7E−13
1202	PS (39:2)	PS	C_45_ H_82_ O_10_ N_1_ P_1_	1.56041	0.01844
200	PE (16:0p/18:1)	PE	C_39_ H_74_ O_7_ N_1_ P_1_	1.51739	0.00018
574	LPC (18:0)	LPC	C_26_ H_54_ O_7_ N_1_ P_1_	1.48342	8.8E−07
600	PA (26:3/20:4)	PA	C_49_ H_81_ O_8_ P_1_	1.48025	0.00034
127	TG (16:1/12:0/18:2)	TG	C_49_ H_88_ O_6_	1.455	1.1E−07
1274	PE (39:1)	PE	C_44_ H_86_ O_8_ N_1_ P_1_	1.4499	4.9E−06
105	DG (18:0/18:1)	DG	C_39_ H_74_ O_5_	1.44806	0.00029
517	TG (16:0/16:1/16:1)	TG	C_51_ H_94_ O_6_	1.44152	5.9E−07
396	TG (18:2/18:2/18:2)	TG	C_57_ H_98_ O_6_	1.42192	6.1E−14
910	TG (18:0/18:1/18:3)	TG	C_57_ H_102_ O_6_	1.40475	2.3E−05
166	TG (18:2/17:1/18:2)	TG	C_56_ H_98_ O_6_	1.40077	5E−12
155	TG (18:1/18:2/22:1)	TG	C_61_ H_110_ O_6_	1.397	1.1E−08
148	TG (19:1/18:1/18:2)	TG	C_58_ H_104_ O_6_	1.3943	1.7E−05
644	PA (24:3/20:4)	PA	C_47_ H_77_ O_8_ P_1_	1.3811	2.4E−05
1060	PC (34:1e)	PC	C_42_ H_84_ O_7_ N_1_ P_1_	1.38093	2.9E−11
1089	PS (39:4)	PS	C_45_ H_78_ O_10_ N_1_ P_1_	1.37928	4.6E−05
972	PC (38:3)	PC	C_46_ H_86_ O_8_ N_1_ P_1_	1.37474	3.8E−09
1318	PEt (42:7)	PEt	C_47_ H_77_ O_8_ P_1_	1.35997	5.1E−06
418	PE (20:1p/20:4)	PE	C_45_ H_78_ O_7_ N_1_ P_1_	1.34442	6.8E−13
37	TG (16:0/14:0/16:1)	TG	C_49_ H_92_ O_6_	1.33804	0.04641
637	SM (d17:0/16:0) + CH3COO	SM	C_40_ H_82_ O_8_ N_2_ P_1_	1.32193	3.6E−06
691	PG (18:0/18:1)	PG	C_42_ H_79_ O_10_ P_1_	1.30451	0.00017
6	DG (16:1/18:2)	DG	C_37_ H_66_ O_5_	1.29582	5.4E−05
135	TG (18:1/18:1/20:3)	TG	C_59_ H_104_ O_6_	1.28907	8.3E−05
167	TG (18:1/18:1/20:4)	TG	C_59_ H_102_ O_6_	1.28051	0.01013
195	PE (18:0p/22:5)	PE	C_45_ H_78_ O_7_ N_1_ P_1_	1.25559	0.00728
478	PE (18:2p/22:6)	PE	C_45_ H_72_ O_7_ N_1_ P_1_	1.24177	1.3E−15
850	SM (d18:1/24:0)	SM	C_47_ H_95_ O_6_ N_2_ P_1_	1.21797	4.3E−08
75	TG (18:1/18:2/24:1)	TG	C_63_ H_114_ O_6_	1.21011	3.6E−13
219	PE (20:0p/22:6)	PE	C_47_ H_80_ O_7_ N_1_ P_1_	1.18724	9.3E−14
387	TG (14:0/18:2/18:2)	TG	C_53_ H_94_ O_6_	1.18626	1.2E−08
571	LPC (16:0)	LPC	C_24_ H_50_ O_7_ N_1_ P_1_	1.1861	0.00037
713	CL (18:2/18:2/18:2/20:4)	CL	C_83_ H_140_ O_17_ P_2_	1.16612	5.1E−13
49	TG (18:1/17:1/18:1)	TG	C_56_ H_102_ O_6_	1.16208	0.00191
279	TG (15:0/16:1/18:2)	TG	C_52_ H_94_ O_6_	1.16013	4.5E−10
100	PS (18:0/20:4)	PS	C_44_ H_76_ O_10_ N_1_ P_1_	1.15838	0.00539
324	PE (16:0p/20:3)	PE	C_41_ H_74_ O_7_ N_1_ P_1_	1.14601	7.6E−08
563	PE (18:1/22:5)	PE	C_45_ H_76_ O_8_ N_1_ P_1_	1.14229	4.7E−09
625	TG (18:1/12:0/18:2)	TG	C_51_ H_92_ O_6_	1.13295	9E−09
169	TG (18:1/18:1/22:5)	TG	C_61_ H_104_ O_6_	1.122	0.00287
1229	PEt (44:8)	PEt	C_49_ H_79_ O_8_ P_1_	1.11401	0.02262
101	PS (18:0/22:4)	PS	C_46_ H_80_ O_10_ N_1_ P_1_	1.11354	1.5E−05
66	TG (18:1/18:1/21:0)	TG	C_60_ H_112_ O_6_	1.09968	1.3E−07
636	PA (22:6/23:3)	PA	C_48_ H_75_ O_8_ P_1_	1.09608	0.00017
773	TG (17:0/17:0/18:3)	TG	C_55_ H_100_ O_6_	1.05744	6.7E−05
1337	dMePE (37:6)	dMePE	C_44_ H_74_ O_8_ N_1_ P_1_	1.05539	5.7E−15
151	TG (18:1/12:0/14:0)	TG	C_47_ H_88_ O_6_	1.05222	0.00569
199	PS (18:0/18:2)	PS	C_42_ H_76_ O_10_ N_1_ P_1_	1.04951	0.00029
954	PC (32:1)	PC	C_40_ H_78_ O_8_ N_1_ P_1_	1.03542	0.00024
583	PC (18:0/18:1)	PC	C_44_ H_86_ O_8_ N_1_ P_1_	1.03295	6.7E−12
831	TG (18:0/18:1/24:1)	TG	C_63_ H_118_ O_6_	1.0111	1.6E−07
693	SM (d17:0/18:1) + CH3COO	SM	C_42_ H_84_ O_8_ N_2_ P_1_	1.00985	0.01846
997	SM (d36:1)	SM	C_41_ H_83_ O_6_ N_2_ P_1_	1.00855	1.5E−07

**Table 2:** The details of the 20 most differentially expressed lipids (sorted by VIP)

ID	Lipid	Class	Formula	VIP	*P*-value
89	PE (18:0/20:4)	PE	C_43_ H_76_ O_8_ N_1_ P_1_	8.06556	1.6E−13
91	PE (18:1/20:4)	PE	C_43_ H_74_ O_8_ N_1_ P_1_	6.77589	7.8E−12
53	TG (18:1/18:1/18:1)	TG	C_57_ H10_4_ O_6_	6.6631	4.9E−09
55	TG (18:1/18:2/18:2)	TG	C_57_ H_100_ O_6_	6.6448	4E−10
1232	PS (37:1)	PS	C_43_ H_80_ O_10_ N_1_ P_1_	6.3032	1.3E−10
93	PE (16:0p/22:6)	PE	C_43_ H_72_ O_7_ N_1_ P_1_	5.94693	1.3E−14
52	TG (18:0/18:1/18:1)	TG	C_57_ H_106_ O_6_	5.18991	0.0007
46	TG (16:0/18:1/18:1)	TG	C_55_ H_102_ O_6_	5.0121	1.4E−08
42	TG (16:1/16:1/18:2)	TG	C_53_ H_94_ O_6_	4.9633	5.1E−13
62	TG (20:0/18:1/18:1)	TG	C_59_ H_110_ O_6_	4.91587	6.3E−12
956	PC (34:1)	PC	C_42_ H_82_ O_8_ N_1_ P_1_	4.8359	3E−13
51	TG (18:0/18:0/18:1)	TG	C_57_ H_108_ O_6_	4.34905	5.3E−07
615	PA (24:2/20:4)	PA	C_47_ H_79_ O_8_ P_1_	4.25154	1.5E−10
963	PC (36:1)	PC	C_44_ H_86_ O_8_ N_1_ P_1_	3.98438	3.9E−13
85	LPE (20:4)	LPE	C_25_ H_42_ O_7_ N_1_ P_1_	3.92548	5.8E−05
704	PA (26:2/20:3)	PA	C_49_ H_85_ O_8_ P_1_	3.88784	6.9E−16
87	PE (16:0p/20:4)	PE	C_41_ H_72_ O_7_ N_1_ P_1_	3.77053	3.3E−07
165	TG (16:1/14:0/18:2)	TG	C_51_ H_92_ O_6_	3.72125	7.4E−08
132	TG (16:0/18:2/18:2)	TG	C_55_ H_98_ O_6_	3.54014	0.01038
115	LPE (22:6)	LPE	C_27_ H_42_ O_7_ N_1_ P_1_	3.43674	0.00013

### Metabolic pathway enrichment analysis

**Fig. 5:**
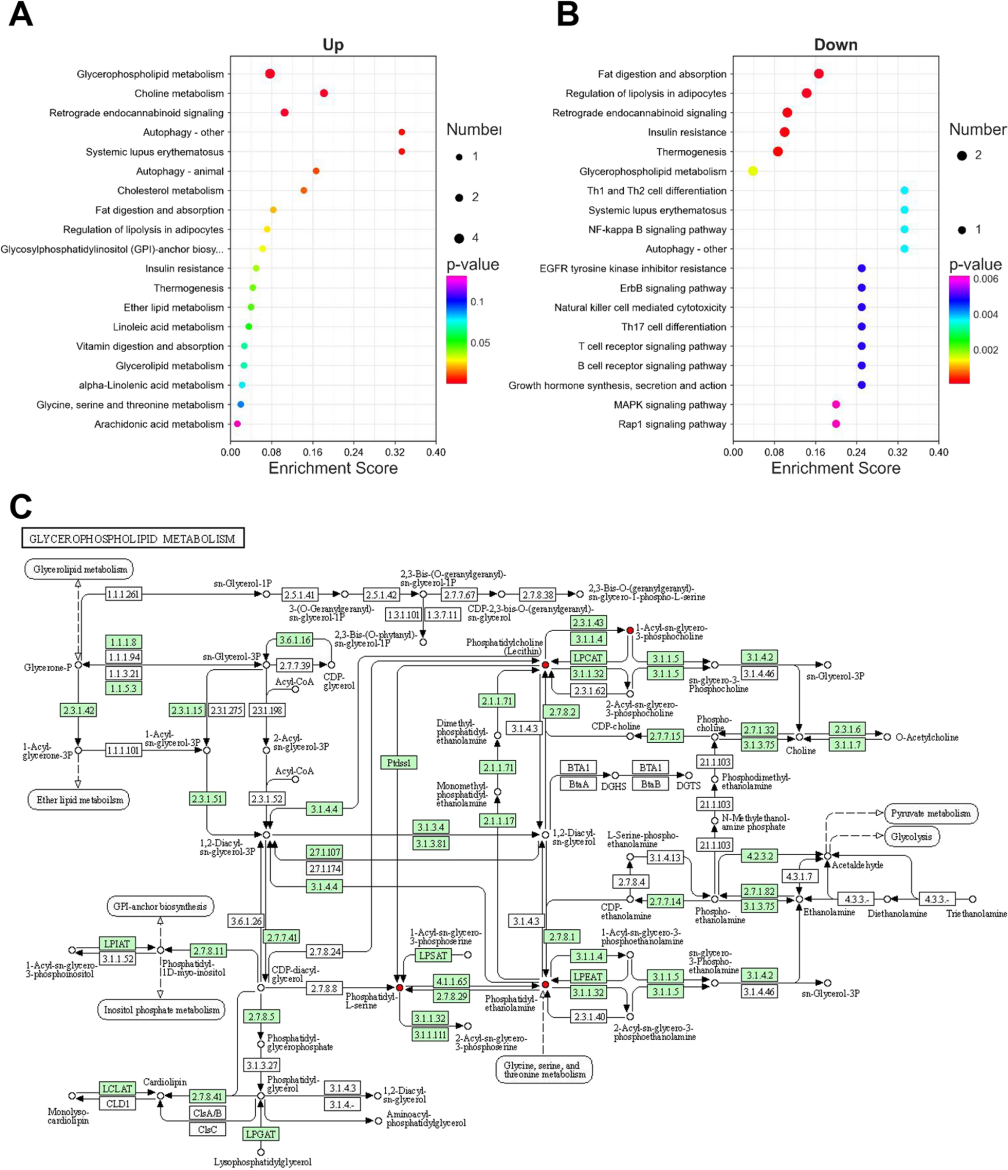
Metabolic pathway enrichment analysis. **A** KEGG pathway analysis of lipids with increased levels. **B** KEGG pathway analysis of lipids with decreased levels. **C** Analysis of individual glycerophospholipid pathways. KEGG, Kyoto Encyclopedia of Genes and Genomes

KEGG pathway enrichment analysis (http://www.kegg.jp/orhttp://www.genome.jp/kegg/) was used to predict metabolic pathways based on the lipids with increased and decreased levels. As shown in Fig. 5A, the lipids with increased levels were mainly associated with glycerophospholipid metabolism, choline metabolism, retrograde endocannabinoid signaling, autophagy-other, systemic lupus erythematosus, autophagy-animal and cholesterol metabolism. Interestingly, the lipids with decreased levels were mainly associated with fat digestion and absorption, regulation of lipolysis in adipocytes, retrograde endocannabinoid signaling, insulin resistance, thermogenesis, glycerophospholipid metabolism and Th1 and Th2 cells differentiation (Fig. 5B). These results indicate that lipid metabolism imbalance, autophagy and inflammatory responses may be involved in the mechanism underlying atherosclerosis. The above results showed that glycerophospholipid metabolism was significantly enriched in both the activated and inhibited KEGG pathways, which attracted our attention. Further analysis of individual glycerophospholipid pathways revealed that some lipids, including phosphatidyl-L-serine, phosphatidyl-ethanolamine, phosphatidylcholine (Lecithin) and 1-acyl-sn-glycero-3-phosphocholine, exhibited increased levels, which may play a critical function in the occurrence or progression of AS (Fig. 5C). Moreover, IPA network pathway analysis showed that differentially expressed lipids were closely related to NF-κB signaling, PI3K/AKT signaling and JAK/STAT signaling (Additional file 3: Fig. S3).

### Lipid composition analysis

The types of the 131 lipid components are shown in Additional file 4: Fig. S4, including TG (n = 53), PE (n = 24), (PC (n = 10), PS (n = 10), SM (n = 7), phosphatidic acid (PA, n = 7), and other (n = 20) lipids. In addition, we showed changes in the expression levels of various lipids, including TG, PE, PC, PS, PA, SM and other lipids, in the ND and HFD groups in the form of a chromatic diagram (Additional file 4: Fig. S4). Interestingly, the levels of all lipids in the PC and LPC classes were significantly increased in the HDF group, which may play a nonnegligible effect in the onset and process of atherosclerosis.

**Fig. 6:**
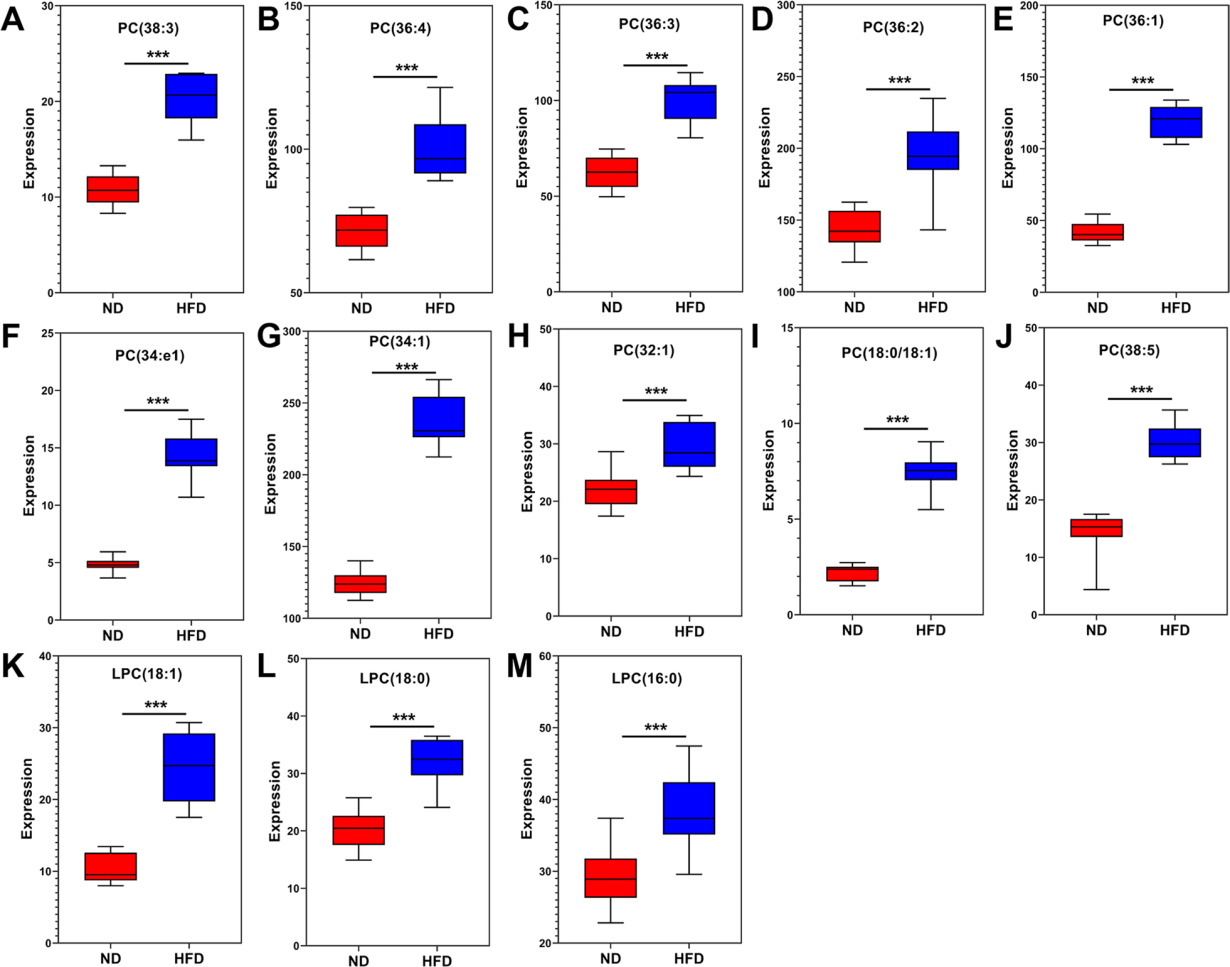
The expression levels of PCs and LPCs. To explore the role of PC and LPC in atherosclerosis, we further analyzed the levels of 10 PCs and 3 LPCs, which exhibited significantly increased levels in the HFD group. **A** PC (38:3), **B** PC (36:4), **C** PC (36:3), **D** PC (36:2), **E** PC (36:1), **F** PC (34:1e), **G** PC (34:1), **H** PC (32:1), **I** PC (18:0/18:1), **J** PC (38:5), **K** LPC (18:1), **L** LPC (18:0), **M** LPC (16:0). N = 10 per group. All data are presented as the means ± SEMs. ***P < 0.001 versus the ND group. PC, phosphatidylcholine; LPC, lysophosphatidylcholine; ND, normal diet; HFD, high-fat diet

As reported, PCs [24, 25] and LPCs [26] are closely related to atherosclerosis. To explore the role of PC and LPC in atherosclerosis, we further analyzed the expression levels of 10 PCs and 3 LPCs, which exhibited significantly increased levels in the HFD group. The levels of PC, including PC (38:3), PC (36:4), PC (36:3), PC (36:2), PC (36:1), PC (34:1e), PC (34:1), PC (32:1), PC (18:0/18:1), and PC (38:5), are shown in Fig. 6A–J, and those of LPC, including LPC (18:1), LPC(18:0) and LPC (16:0), are shown in Fig. 6K–M.

## Discussion

Dyslipidemia is a major risk factor for atherosclerosis and atherosclerosis-related cardiovascular disease [27]. A consensus has been reached on the conclusion that elevated plasma LDL-cholesterol levels promote AS. LDL lipoproteins penetrate damaged endothelial cells, accumulate in the intima after oxidation, and subsequently recruit monocytes to endothelial cells, causing monocytes to differentiate into macrophages and form foam cells, initiating and accelerating the process of atherosclerosis [9]. Unlike cholesterol, triglycerides are also associated with subclinical atherosclerosis and vascular inflammation [28]. In this study, the lipid profile of the blood vessels in AS mice was investigated through lipidomic technology, and 131 differential lipids were identified in the thoracic aorta of ND- and HFD-treated *ApoE*^*−/−*^ mice, including TG, PE, and PC. Unlike in the ND group, the top 20 lipid components included PE (18:0/20:4), PE (18:1/20:4), TG (18:1/18:1/18:1), TG (18:1/18:2/18:2), PS (37:1), PE (16:0p/22:6), TG (18:0/18:1/18:1), TG (16:0/18:1/18:1), TG (16:1/16:1/18:2), TG (20:0/18:1/18:1), PC (34:1), TG (18:0/18:0/18:1), PA (24:2/20:4), PC (36:1), LPE (20:4), PA (26:2/20:3), PE (16:0p/20:4), TG (16:1/14:0/18:2), TG (16:0/18:2/18:2), and LPE (22:6) in the AS group.

Recently, omics approaches have been used to improve the understanding of the pathogenesis of AS. For example, Wang et al. [17] used ultraperformance liquid chromatography coupled with quadrupole time-of-flight high-definition mass spectrometry (UPLC-Q-TOF/MS) to identify 17 lipid components closely related to the occurrence and development of AS in the serum of *ApoE*^*−/−*^ mice. The lipids included DAG (14:0/18:2), PC (18:0/20:1), LPC (18:2), LPC (20:2), PC (18:2/22:6), PE (18:0/18:2), SM (D18:1/16:0), LPE (20:1), TAG48:1-FA12:0, TAG48:1-FA14:0, TAG49:2-FA17:0, TAG52:6-FA16:0, TAG53:3FA16:0, TAG53:3-FA17:0, TAG54:6-FA20:5, TAG54:2-FA16:0, and TAG54:0-FA18:0. Jihan et al. [18] developed a new Multi-ABLE method to analyze small tissue samples (< 5 mg) and determined the aortic lipid profile in *ApoE*^*−/−*^ mice. The results showed that 52 lipid molecules exhibited increased abundance in lesion-containing tissue and included SM (n = 7), phospholipids (n = 24) and CL (n = 3). The results regarding the lipids identified by lipidomics in thoracic aorta of *ApoE*^*−/−*^ mice in this article and those in previous studies are inconsistent, which suggested the data from vascular lipidomics should be further investigated to provide an in-depth understanding of the complex pathogenesis of AS.

Recent studies have shown that glycerol phospholipid metabolism plays a critical role in the pathogenesis of AS, and disordered glycerol phospholipid metabolism directly affects the process of AS [29, 30]. In our study, the KEGG enrichment analysis of the lipids that were identified to exhibit increased and decreased levels revealed enrichment in glycerol phospholipid metabolism, indicating that glycerol phospholipid metabolism may be an initiating factor; these results are conducive to further elucidating the pathogenesis of AS. As reported, the major classes of glycosphingolipids, comprising phosphatidylcholine (PC), PE, and PS, are closely related to atherosclerosis. In all nucleated mammalian cells, PC and PE are the most abundant phospholipids in cell membranes [31, 32]. PC is mainly synthesized by the CDP-choline pathway [33]. In an additional pathway, under the condition of S-adenosylmethionine (AdoMet) as a methyl donor, PE can synthesize PC through three sequential methylation reactions [34]. Impaired phosphatidylcholine biosynthesis has been reported to reduce atherosclerosis in *ApoE*^*−/−*^ mice [24]. Exteriorized PS and PE residues on the cell membrane in apoptotic cells trigger rapid phagocytosis and contribute to plaque vulnerability [35–37]. Among patients with CAD, SMs are directly related to serum levels of total cholesterol and lipoproteins, which suggests that sphingolipids play a critical role in modulating lipid serum levels. The results above highlight the importance of establishing new goals in the glycerol metabolic pathway for antiatherogenic therapies [38].

In addition, we found that the levels of 10 PCs from HFD-treated *ApoE*^*−/−*^ mice were significantly higher than those in the ND group, including PC (38:3), PC (36:4), PC (36:3), PC (36:2), PC (36:1), PC (34:1e), PC (34:1), PC (32:1), PC (18:0/18:1), and PC (38:5). Phosphatidylcholine (PC) is a subclass of glycerophospholipids with choline as the head group. The physiological functions of PC include membrane integrity/stability, cell proliferation and survival, fuel and energy storage/source, glycerophospholipid metabolism and cell signaling [31, 39]. It has been reported that the levels of PCs (16:2/22:6, 16:2/20:5, 15:0/18:2) were decreased in stroke patients [40]. In contrast, PC (16:0/20:4) levels were significantly higher in the serum of patients with severe calcific coronary artery disease than in patients with noncalcific coronary artery disease, while PC(18:2/18:2) and PC(36:3) levels had the opposite trend [41].

Lysophosphatidylcholine (LPC) is the main component of oxidized low-density lipoprotein (oxLDL). In cells, saturated LPC (16:0) and PC can be interconverted. Specifically, PC can be cleaved by phospholipase A2 (PLA2) to form LPC [42, 43], and LPC can be converted to PC by lysophosphatidylcholine acyltransferase (LPCAT) [44]. In addition, lecithin-cholesterol acyltransferase (LCAT) can transfer fatty acids to free cholesterol to form LPC [44]. LPC damages endothelial cells, induces smooth muscle cell proliferation and lymphocyte and macrophage migration, increases inflammatory mediator production and oxidative stress, and induces cell apoptosis and necrosis, which accelerate the progression of atherosclerosis and atherosclerosis-related cardiovascular diseases [26, 45, 46]. In patients with hypertension, LPCs can be considered potential predictors for further evaluation and validation of early vascular aging (EVA) [47]. As reported, the levels of lysoPC 16:0, 18:0, and 18:1 in human plaques are markedly related to inflammatory mediators, such as monocyte chemoattractant protein-1 (MCP1), interleukin-1β (IL-1β), interleukin-6 (IL-6), and macrophage inflammatory protein-1β (MIP-1β) [48], which suggests that lysoPCs play a key role in plaque inflammation and stability. In our study, we also found that the levels of these three lipids were elevated in the vascular tissue of mice with atherosclerosis, which was consistent with the above research report. This finding indicates that LPC (16:0), LPC (18:0) and LPC (18:1) may participate in the process of atherosclerosis and be closely related to vascular injury.

Although we revealed the lipidomic profiling of vascular tissue of atherosclerotic mice by LC‒MS/MS technology and identified some differentially expressed lipid molecules, there are still some limitations. First, due to the continuous development of LC‒MS/MS technology, lipidomics methods are widely used to comprehensively analyze lipids and their interactions with other molecules. However, it is not clear whether all lipids in some biological samples can be comprehensively detected and accurately identified due to the complex composition of lipids. It is also difficult to conduct accurate localization analysis on the detected lipids. Therefore, the development of new instruments and new bioinformatics schemes, such as ion mobility mass spectrometry (IM-MS) [49], NanoLC‒MS [50], AFADESI-MSI [51]and spatial metabolomics analysis [52], may bring new insights into the application of metabolomics and lipidomics in the future. Second, our study suggested that PCs and LPCs may have a strong association with atherosclerosis, but the causal relationship between them needs to be further clarified. In addition, we observed an interesting phenomenon. While confirming the successful establishment of the atherosclerosis model, there was no significant difference in the TG levels in the serum between mice in the HFD group and those in the ND group. More interestingly, our lipidomics spectrum analysis also revealed that the levels of TG lipid molecules were not increased in the HFD group, and the levels were in fact lower. It is possible that the specificity of TG as a marker of AS needs further reference.

## Conclusion

Our findings indicate that in the comprehensive lipid profile in the vascular tissue of atherosclerotic mice, a total of 131 differential lipids were identified, including 57 lipids with levels that were increased in the HFD group and 74 with levels that were decreased. Further analysis revealed that the identified differentially expressed PCs and LPCs exhibited significantly increased levels. We particularly analyzed the details of PCs and LPCs, which exhibited significantly increased levels in AS. Therefore, targeting PCs, LPCs and lipid metabolism might be a novel and effective therapeutic strategy for atherosclerosis.

## Supplementary Information

**Additional file 1: Figure S1**. The base peak chromatograms (BPCs) of positive ions and negative ions. **A** The BPCs of positive ions in the ND group. **B** The BPCs of negative ions in the ND group. **C** BPCs of positive ions in the HFD group. **D** The BPCs of negative ions in the HFD group. BPC, base peak chromatogram; ND, normal diet; HFD, high-fat diet.

**Additional file 2: Figure S2**. The multivariate statistical analysis. **A** PCA analysis. **B** OPLS-DA. PCA, principal component analysis; OPLS-DA, orthogonal partial least squares discriminant analysis.

**Additional file 3: Figure S3**. IPA network pathway analysis. The IPA network pathway analysis revealed that these differentially expressed lipids were related to NF-κB signaling, PI3K/AKT signaling, and JAK/STAT signaling. IPA, ingenuity pathway analysis; CP, classical signaling pathway.

**Additional file 4: Figure S4**. Chromatogram analysis of various lipid components. **A** A total of 131 differential lipids, including 53 TG (40.46%), 24 PE (18.32%), 10 PC (7.63%), 10 PS (7.63%), 7 SM (5.34%), 7 PA (5.34%) and 20 other lipids (15.27%), were identified. **B** TG, **C** PE, **D** PC, **E** PS, **F** PA, **G** SM, **H** Other lipids. TG, triglyceride; PE, phosphatidylethanolamine; PC, phosphatidylcholine; PS, phosphatidylserine; PA, phosphatidic acid; SM, sphingomyelin; LPE, lysophosphatidylethanolamine; LPC, lysophosphatidylcholine; DG, diglyceride; CL, cardiolipin; PG, phosphatidylglycerol; PEt, phosphatidylethanol; dMePE, dimethyl phosphatidylethanolamine.

## Data Availability

Not applicable.

## Abbreviations

AS: Atherosclerosis
ND: Normal diet
HFD: High-fat diet
LDL: Low-density lipoprotein
TC: Total cholesterol
TG: Triglyceride
UHPLC-Q Exactive MS: Ultrahigh-performance liquid chromatography plus Q-Exactive mass spectrometry
PC: Phosphocholine
LPC: Lysophosphocholine
IDL: Intermediate-density lipoprotein
LC‒MS: Liquid chromatography coupled to MS
SM: Sphingomyelin
CE: Electrophoresis
PE: Phosphatidylethanolamine
PS: Phosphatidylserine
Cer: Ceramide
LPE: Lysophosphatidylethanolamine
CE: Cholesteryl esters
DAG: Diacylglycerol
ESI: Electrospray ionization
PA: Phosphatidic acid
IPA: Ingenuity pathway analysis
PCA: Principal component analysis
OPLS-DA: Orthogonal partial least squares-discriminant analysis
PLS-DA: Partial least squares-discriminant analysis
VIP: Variable importance of projection
KEGG: Kyoto Encyclopedia of Genes and Genomes
BPCs: Base peak chromatograms
oxLDL: Oxidized low-density lipoprotein
PLA2: Phospholipase A2
LPCAT: Lysophosphatidylcholine acyltransferase
LCAT: Lecithin-cholesterol acyltransferase
EVA: Early vascular aging
MCP1: Monocyte chemoattractant protein-1
IL-1β: Interleukin-1β
IL-6: Interleukin-6
MIP-1β: Macrophage inflammatory protein-1β
