# Supplementary figures and images for: Vascular lipidomics analysis reveales increased levels of phosphocholine and lysophosphocholine in atherosclerotic mice
Source: Nutr Metab (Lond). 2023 Jan 4;20:1. doi: 10.1186/s12986-022-00723-y (PMC9811766; doi:10.1186/s12986-022-00723-y)

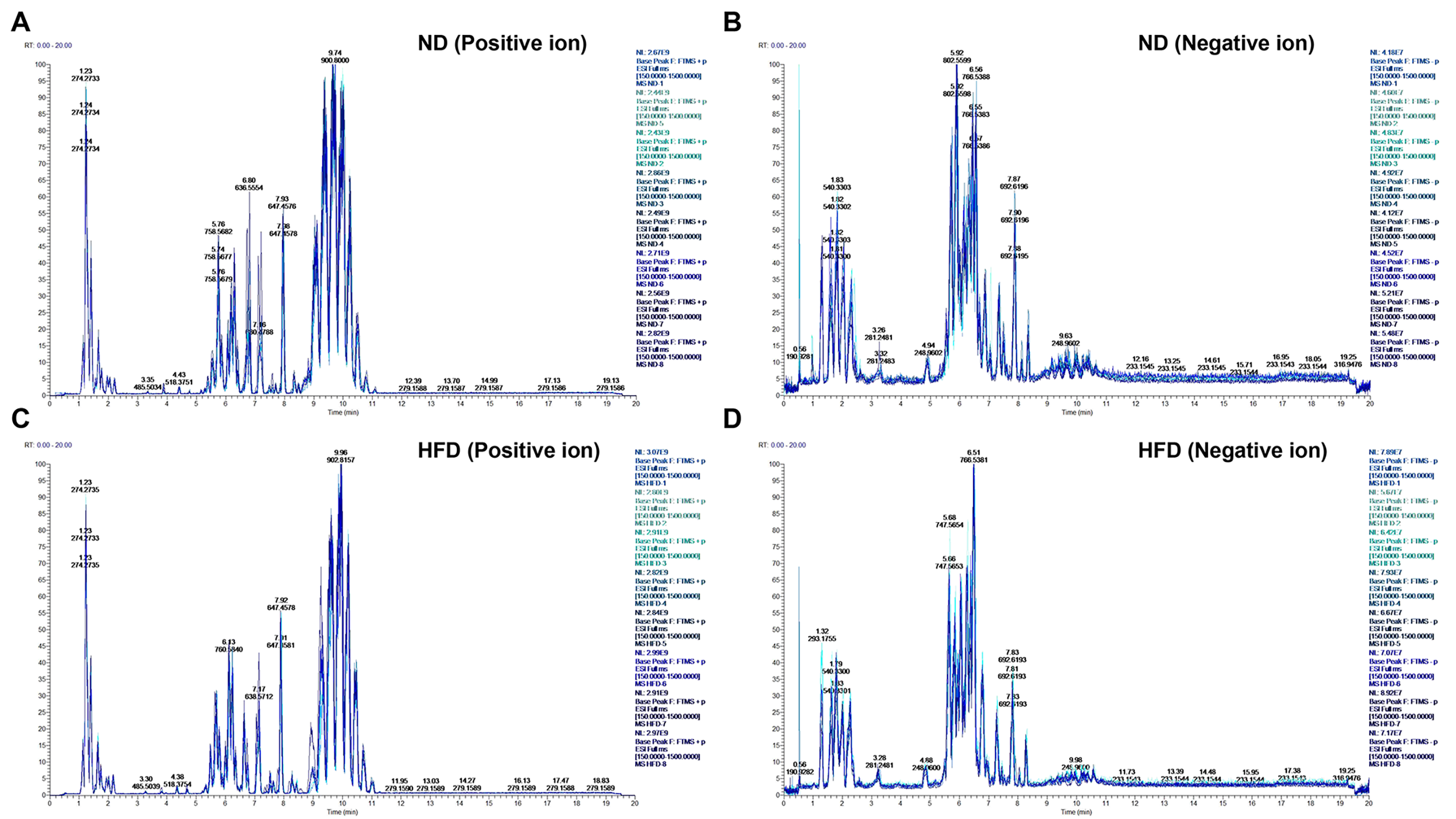

Supplement: Supplementary file 1 — Additional file 1: Figure S1. The base peak chromatograms (BPCs) of positive ions and negative ions. A The BPCs of positive ions in the ND group. B The BPCs of negative ions in the ND group. C BPCs of positive ions in the HFD group. D The BPCs of negative ions in the HFD group. BPC, base peak chromatogram; ND, normal diet; HFD, high-fat diet. [file 12986_2022_723_MOESM1_ESM.tif]

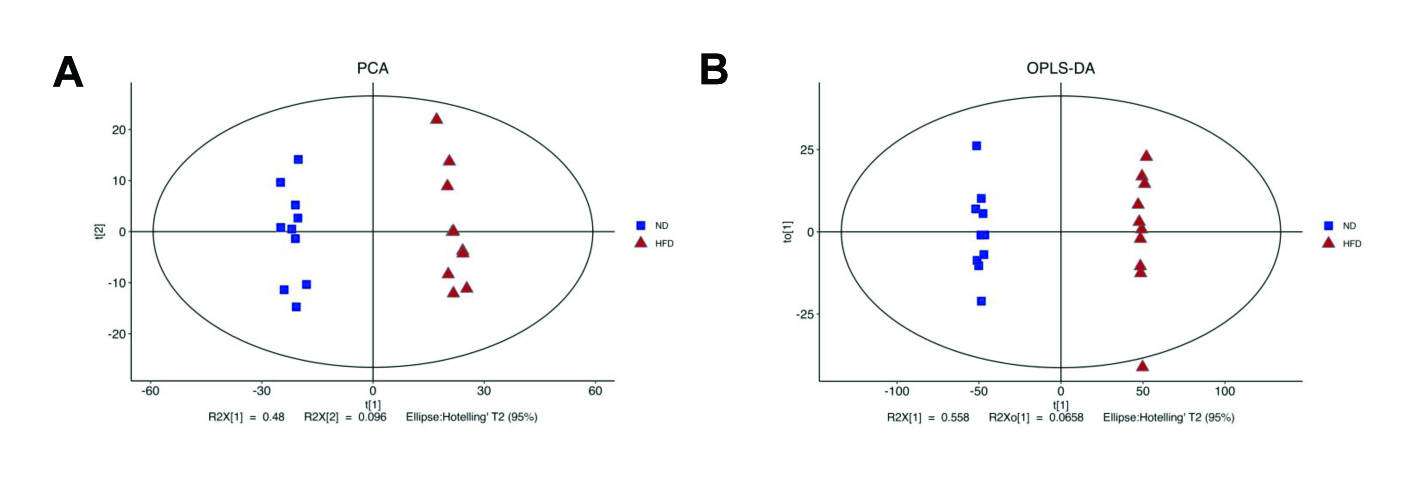

Supplement: Supplementary file 2 — Additional file 2: Figure S2. The multivariate statistical analysis. A PCA analysis. B OPLS-DA. PCA, principal component analysis; OPLS-DA, orthogonal partial least squares discriminant analysis. [file 12986_2022_723_MOESM2_ESM.tif]

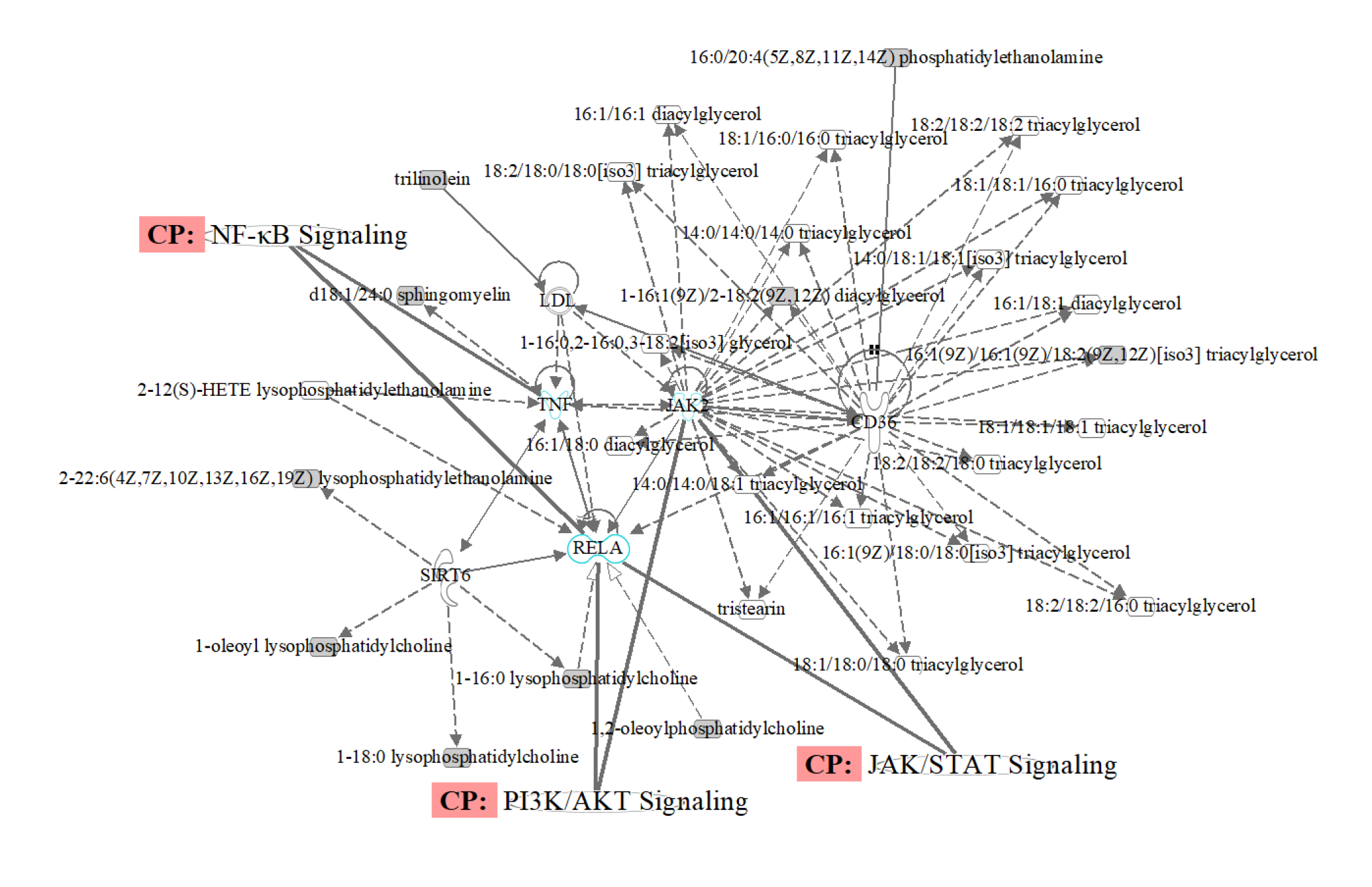

Supplement: Supplementary file 3 — Additional file 3: Figure S3. IPA network pathway analysis. The IPA network pathway analysis revealed that these differentially expressed lipids were related to NF-κB signaling, PI3K/AKT signaling, and JAK/STAT signaling. IPA, ingenuity pathway analysis; CP, classical signaling pathway. [file 12986_2022_723_MOESM3_ESM.tif]

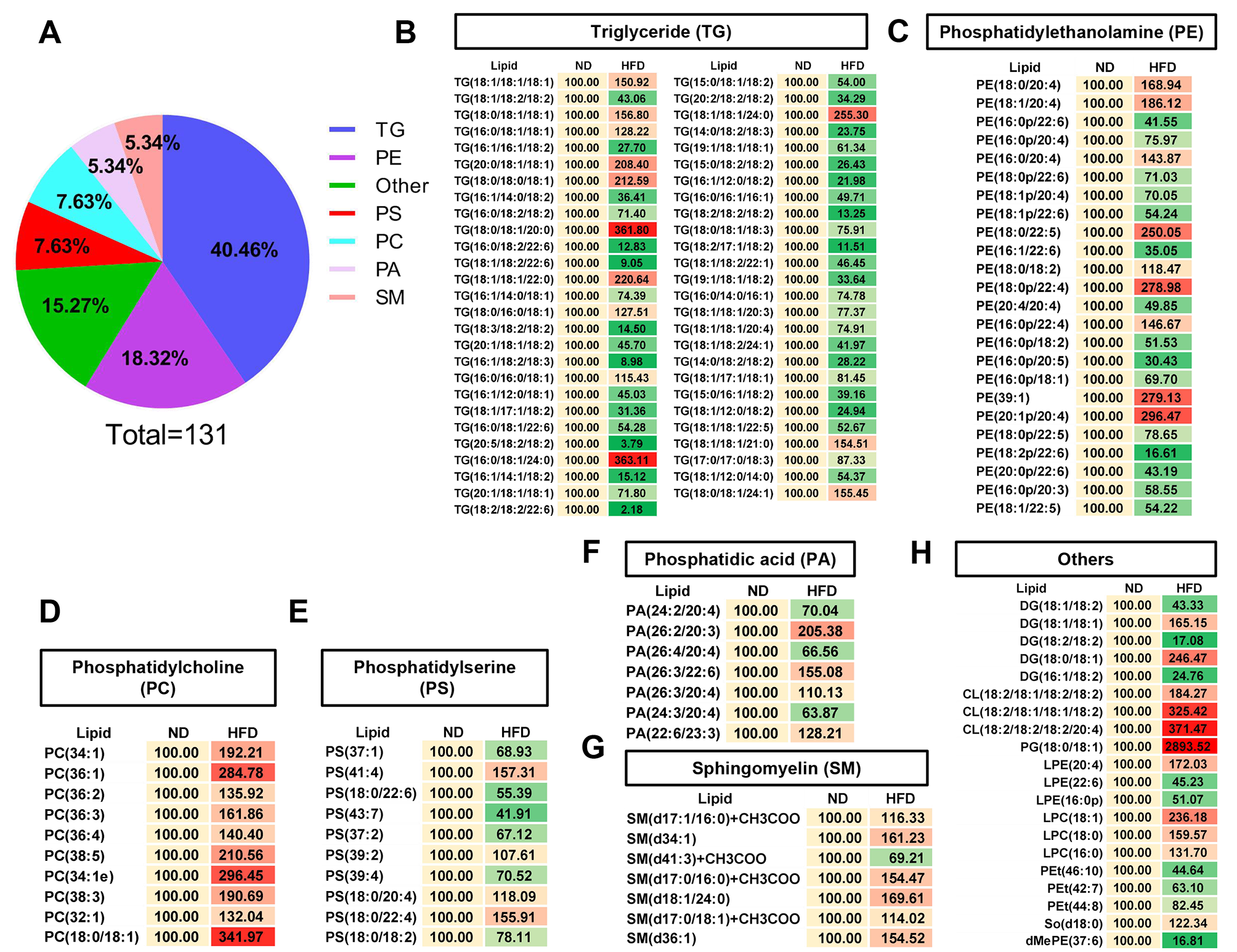

Supplement: Supplementary file 4 — Additional file 4: Figure S4. Chromatogram analysis of various lipid components. A A total of 131 differential lipids, including 53 TG (40.46%), 24 PE (18.32%), 10 PC (7.63%), 10 PS (7.63%), 7 SM (5.34%), 7 PA (5.34%) and 20 other lipids (15.27%), were identified. B TG, C PE, D PC, E PS, F PA, G SM, H Other lipids. TG, triglyceride; PE, phosphatidylethanolamine; PC, phosphatidylcholine; PS, phosphatidylserine; PA, phosphatidic acid; SM, sphingomyelin; LPE, lysophosphatidylethanolamine; LPC, lysophosphatidylcholine; DG, diglyceride; CL, cardiolipin; PG, phosphatidylglycerol; PEt, phosphatidylethanol; dMePE, dimethyl phosphatidylethanolamine. [file 12986_2022_723_MOESM4_ESM.tif]
